# Validation of differentially methylated microRNAs identified from an epigenome-wide association study; Sanger and next generation sequencing approaches

**DOI:** 10.1186/s13104-018-3872-x

**Published:** 2018-10-29

**Authors:** Laura J. Smyth, Alexander P. Maxwell, Katherine A. Benson, Jill Kilner, Gareth J. McKay, Amy Jayne McKnight

**Affiliations:** Genetic Epidemiology Research Group, Centre for Public Health, Queen’s University of Belfast, Belfast, UK

**Keywords:** Diabetes, Epigenetic, EWAS, Kidney, Methylation, microRNA, NGS, Renal, Sequencing, SNP

## Abstract

**Objectives:**

Altered DNA methylation and microRNA profiles are associated with diabetic kidney disease. This study compared different sequencing approaches to define the genetic and epigenetic architecture of sequences surrounding microRNAs associated with diabetic kidney disease.

**Results:**

We compared Sanger and next generation sequencing to validate microRNAs associated with diabetic kidney disease identified from an epigenome-wide association study (EWAS). These microRNAs demonstrated differential methylation levels in cases with diabetic kidney disease compared to controls with long duration of type 1 diabetes and no evidence of kidney disease (P_adjusted_ < 10^−5^). Targeted next generation sequencing analysis of genomic DNA and matched cell-line transformed DNA samples identified four genomic variants within the microRNAs, two within *miR*-*329*-*2* and two within *miR*-*429*. Sanger sequencing of genomic DNA replicated these findings and confirmed the altered methylation status of the CpG sites identified by the EWAS in bisulphite-treated DNA. This investigation successfully fine-mapped the genetic sequence around key microRNAs. Variants have been detected which may affect their methylation status and methylated CpG sites have been confirmed. Additionally, we explored both the fidelity of next generation sequencing analysis and the potential efficacy of cell-line transformed DNA samples in place of finite patient samples in discovery genetic and epigenetic research.

**Electronic supplementary material:**

The online version of this article (10.1186/s13104-018-3872-x) contains supplementary material, which is available to authorized users.

## Introduction

Diabetic kidney disease (DKD) is a major complication of diabetes mellitus (DM) and the most common cause of chronic kidney disease (CKD) worldwide [1–3]. DKD is also associated with an increased risk of cardiovascular mortality [4, 5]. DKD has a complex aetiology, yet individual risk is greatly influenced by genetic predisposition [6].

Advances in next generation sequencing (NGS) technologies and analytical approaches have resulted in more cost-effective sequencing [7], accelerating the rate of genetic research [8]. However, NGS costs are still prohibitive for many laboratories, limiting its utility in large-scale studies of the methylome using high-density arrays [9, 10].

Several genome-wide association studies (GWAS) and meta-analyses have been undertaken to detect common genetic variants associated with DKD. These investigations identified single nucleotide polymorphisms (SNPs) associated with DKD including *FRMD3* [11], *CARS* [11], *ACACB* [12], *AFF3* [13], *CDCA7* [14], *CUBN* [15] and *EPO* [16] genes.

Epigenetic modifications influence both DNA and RNA regulation without altering the underlying sequence and may contribute to the inherited predisposition of DKD [17, 18]. DNA methylation is significantly altered in DM with higher levels of methylation reported in individuals with DKD [19]. MicroRNAs (miRNAs) are small highly conserved non-coding RNA molecules that act as epigenetic modifiers in the regulation of many protein-coding genes [20, 21] and gene expression [22]. MiRNAs play a vital role in many diseases [23].

Induction of miRNAs in renal cells is associated with accumulation of extracellular matrix proteins implicated in kidney fibrosis and glomerular dysfunction [21]. Several miRNAs have been reported previously in association with DKD including *miR*-*135a* [24], *miR*-*200b* [25] and *miR*-*377* [26]. MiRNAs may represent biomarkers for this disease but further mechanistic studies are required to elucidate their effects.

This study compared sequencing approaches to investigate differentially methylated miRNAs associated with DKD identified from an epigenome-wide association study (EWAS). The aims were to determine genetic variants and epigenetic marks in the miRNAs associated with DKD and their surrounding sequences, and to perform a direct comparison of the results between blood-derived genomic DNA (gDNA) and DNA from Epstein-Barr virus transformed cell-lines derived from the same participants. This provided an opportunity to evaluate the more readily available transformed cell-line DNA samples as a proxy for the finite supply of gDNA.

## Main text

### Methods

#### Sample cohort

All participants were of Caucasian ancestry from the UK or ROI and provided written informed consent for research. DNA was extracted from whole blood using the salting out method, normalised following PicoGreen quantitation, and frozen in multiple aliquots. Cell-line DNA was obtained following Epstein-Barr virus transformation of participants’ lymphocytes into cell lines performed by the European Collection of Authenticated Cell Cultures (ECACC) [27].

Participants were part of the All Ireland-Warren 3-Genetics of Kidneys in Diabetes (GoKinD) UK Collection. Cases (n = 150) were defined as individuals with ≥ 10 years duration of type 1 diabetes (T1D) who had also been diagnosed with DKD defined as hypertension (blood pressure ≥ 135/85 mmHg) and persistent macroalbuminuria (≥ 500 mg/24 h). Diabetic controls (DCs, n = 100) were individuals with ≥ 15 years duration of T1D and no evidence of renal disease on repeat testing. Control subjects all had an estimated glomerular filtration rate (eGFR) > 60 mL/min/m^2^ whereas each case subject had CKD based on presence of persistent macroalbuminuria and eGFR < 60 mL/min/m^2^. Participant characteristics are included within Additional file 1: Table S1.

#### Discovery 450K methylation

Blood-derived gDNA for each individual was bisulphite treated (BST) using the EZ-96 DNA Methylation-Gold™ Kit (Zymo Research, USA).

To assess the methylation status of the cytosine-phosphate-guanine (CpG) sites, the Infinium Human Methylation 450K BeadChip array was used following the manufacturer’s instructions. Cases and controls were randomly distributed across each array. This high throughput platform evaluated individual methylation levels (β values) for each CpG site, ranging from 0 for unmethylated to 1 for complete methylation. Raw methylation data was adjusted for dye bias and quantile normalised as previously reported [28]. Quality control (QC) included evaluation of the bisulphite treatment conversion efficiency, dye specificity, hybridisation, staining and the inclusion of 600 integral negative controls for the EWAS.

The significant methylation values between cases and controls for all probes which passed QC were adjusted for multiple testing using the Benjamini and Hochberg approach [29]. All miRNAs that demonstrated significantly altered levels of DNA methylation (p < ×10^−5^) were selected from our previous EWAS [28] for this validation and fine-mapping study.

#### NGS: targeted DNA sequencing

Targeted NGS analysis was performed for the sequences surrounding the CpG site of interest for each miRNA. Blood-derived gDNA was analysed in 23 DKD cases and 23 DCs. Participant characteristics are included within Additional file 1: Table S2. The gDNA samples were matched to the GoKinD cell-line DNA samples from which they were originally transformed, therefore analysis was conducted for 92 samples for each genomic region.

Target sequences for the five miRNAs were amplified using custom designed primers via a polymerase chain reaction (PCR). DNA fragments were pooled by sizes of approximately 800 base pairs (bp), 400 bp and 200 bp. Optimal primers were designed using Primer3Plus, Vector NTI Advance^®^ (Invitrogen™, USA) and EpiDesigner software. Primers were selected depending on their ability to sufficiently cover the CpG site of interest and have compatible annealing temperature to enable multiplex reactions. Primer sequences are provided in Additional file 1: Table S3 with optimised PCR conditions.

The library preparation was conducted using two Thermo Fisher Scientific protocols. The *Ion Xpress™ Plus gDNA Fragment Library Preparation* protocol (*MAN0009847*, *revision B.0*) was employed where the initial fragments were approximately 800 bp as they required fragmentation using the E-Gel™ SizeSelect™ 2% Agarose Gel to generate 200 bp libraries. For fragments originally of 200 bp and 400 bp, the *Prepare Amplicon Libraries without Fragmentation Using the Ion Plus Fragment Library Kit* protocol (*MAN0006846*, *revision A.0*) was followed.

Following library preparation, the DNA samples were diluted to 26 pM using DNA-free water. The 400 bp libraries were enriched using Thermo Fisher Scientific’s Ion OneTouch™ 2 (OT2) and Enrichment System (ES) (*Ion Personal Genome Machine*^*®*^ (PGM™) *Template OT2 400 Kit manual MAN0007219, revision 3.0*). The 200 bp libraries were enriched and prepared using the Ion Chef™ (*Ion PGM™ IC 200 Kit* manual, *MAN0007661*, *revision A.0*).

Both the Ion 316™ Chip v2 and the Ion 318™ Chip v2 were used to sequence the DNA samples using the Ion PGM™ System (Thermo Fisher Scientific), before the raw data was analysed using Torrent Suite™ Software v4.0.4 and Partek^®^ Genomics Suite^®^ 6.6 software (Partek^®^ Inc., USA). The sequencing reads were aligned to the hg19 reference sequence. SNPs were aligned to dbSNP version 141 and annotated using RefSeq version 2014-07-30. The chromosome viewer was used to visualise the overall sequencing coverage for the region of interest surrounding the top-ranked CpG site for each miRNA.

#### Sanger sequencing: fine mapping and methylation analysis

Forty-six gDNA samples, 23 DKD cases and 23 DCs, were bi-directionally Sanger sequenced using the ABI 3730 Genetic Analyser (Thermo Fisher Scientific). This was completed to enable direct comparisons to be drawn against the NGS variant calls.

Bisulphite treatment of the same samples was performed using the EZ-96 DNA Methylation™ Lightning Kit prior to Sanger sequencing. The resulting data provided the opportunity to assess the methylation status of each CpG site within the fragment.

ContigExpress, a component of Vector NTI Advance^®^ 11.5.2 was used to analyse the Sanger sequencing data and determine accurate SNP calls. DNA sequences were aligned to the GRCh37 reference genome obtained from online resource, Ensembl.

**Fig. 1:**
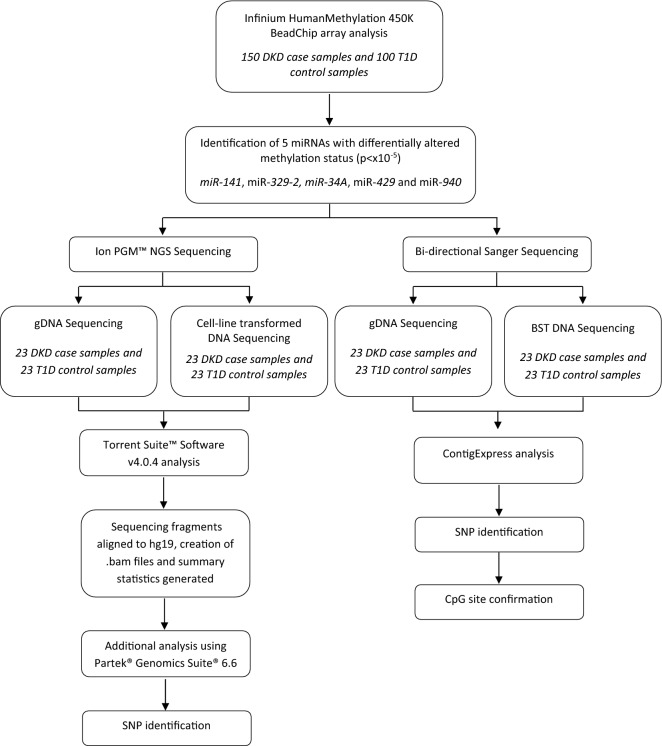
Workflow of analysis methods undertaken in this study. *bam* binary alignment map, *BST* bisulphite-treated, *CpG* cytosine-phosphate-guanine, *DKD* diabetic kidney disease, *gDNA* genomic DNA, *hg* human genome, *NGS* next generation sequencing, *SNP* single nucleotide polymorphism, *T1D* type-1 diabetes mellitus

An overview of the analysis workflow is illustrated in Fig. 1.

### Results

#### Discovery 450K methylation analysis

Methylation status was quantitatively determined (DKD cases n = 150 and DCs n = 100). QC showed that > 99% concordance was observed between all included individuals; r^2^ > 0.98 for each of the sample pairs assessed. In total, 74 CpG sites were determined from the EWAS, five of which were identified with significantly altered β levels from the original EWAS protocol [28]; *miR*-*141*, *miR*-*329*-*2, miR*-*34A*, *miR*-*429* and *miR*-*940* (Additional file 1: Table S4). This manuscript is focused on validation and fine-mapping of these top-ranked miRNAs in individuals with and without DKD.

#### NGS: targeted DNA sequencing

Targeted NGS was performed using the Ion PGM™ for DNA extracted from both whole blood and cell-line DNA. Both the Ion 316™ Chip v2 and the Ion 318™ Chip v2 were used in this analysis which typically generated 1.6 million to 3 million reads (Additional file 2: Figure S1).

**Fig. 2:**
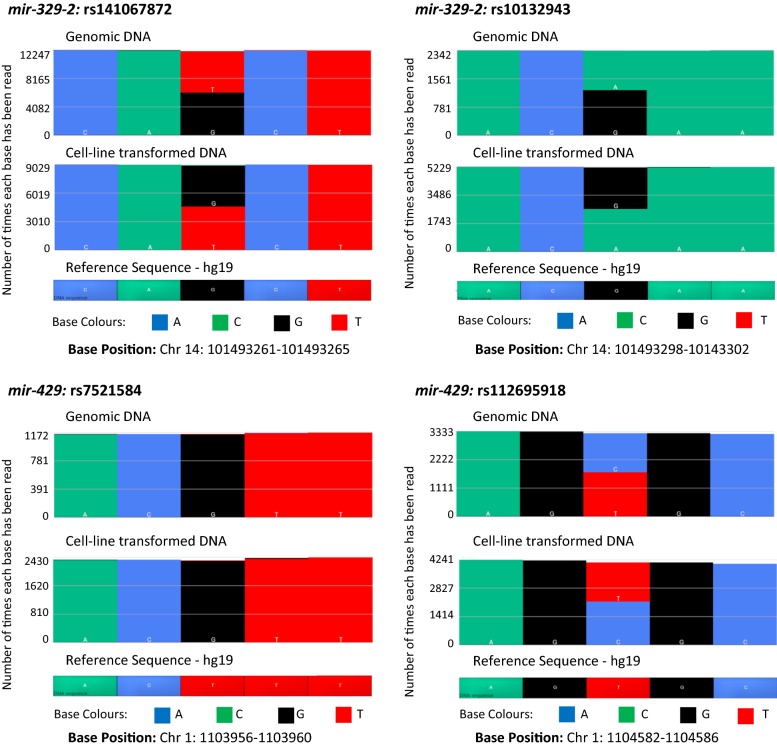
Comparison of matched genomic and cell-line transformed DNA for identified SNPs. Comparison of matched genomic and cell-line transformed DNA for rs141067872, rs10132942 (both *miR*-*329*-*2*), rs7521574 and rs112695918 (both *miR*-*429*) data generated by the Ion PGM™. The matching gDNA and cell-line transformed DNA show consistent results indicated by the base colour patterns in each example. *Chr* chromosome, *hg* human genome

Analysis was completed using Torrent Suite™ Software and Partek^®^ Genomics Suite^®^. Four SNPs were identified which have not previously been associated with DKD, or identified as top-ranked results in GWAS (Fig. 2); two within *miR*-*329*-*2* (rs141067872 and rs10132943) and two within *miR*-*429* (rs7521584 and rs112695918). The frequency distributions for these SNPs are included in Additional file 1: Table S5.

Figure 2 also shows the comparative results of the blood-derived gDNA samples and their complementary cell-line transformed DNA samples, both analysed using the Ion PGM™ (23 DKD cases and 23 DCs). This comparison of matched samples; gDNA compared to cell-line DNA, showed 100% concordance for SNP calls.

#### Sanger sequencing: fine mapping and methylation analysis

**Fig. 3:**
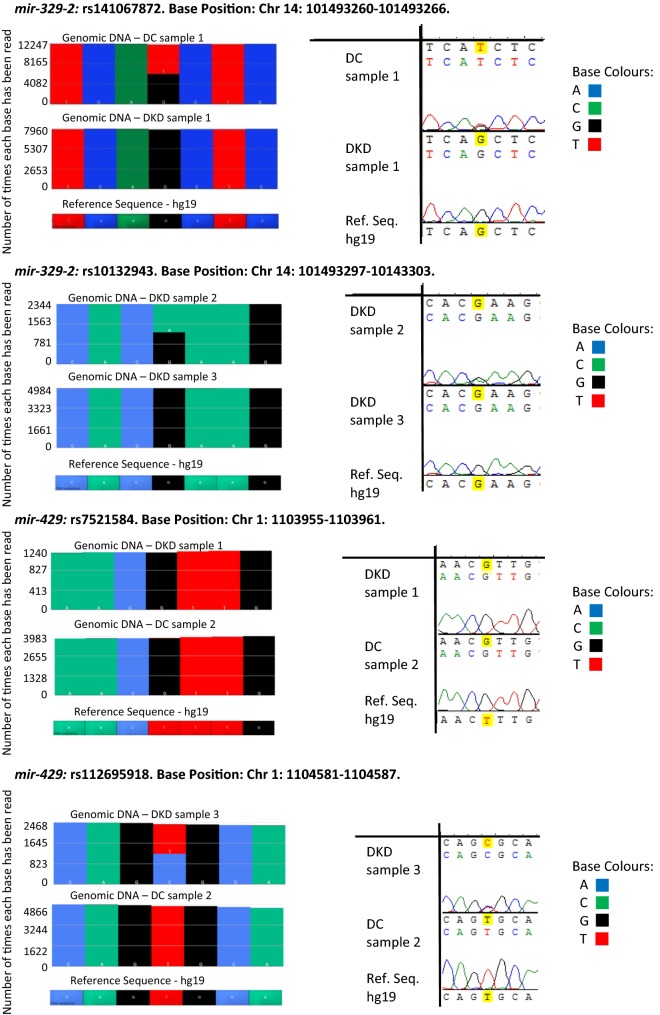
Comparison of SNPs located within *miR*-*329*-*2* and *miR*-*429* identified by targeted NGS and Sanger sequencing. The data generated by both platforms showed consistent results for SNP calls. Ion PGM™ data analysed using Partek Genomics Suite is shown on the left, with the complementary Sanger sequence results shown on the right, for matching genomic DNA samples. *Chr* chromosome, *DC* diabetic control, *DKD* diabetic kidney disease, *hg* human genome, *Ref* reference, *Seq* sequence

To confirm variants identified by NGS, the same primer pairs were used to bi-directionally Sanger sequence matched gDNA samples (23 DKD case and 23 DCs). The variants identified by the NGS approach were confirmed by Sanger sequencing (Fig. 3). The genotype and minor allele frequencies (dbSNP, HapMap-CEU, low coverage panel) determined are detailed in Additional file 1: Table S5, though it is essential to note that not all fragments for all samples were Sanger sequenced successfully.

The gDNA DKD and DC samples were also BST in order to assess the methylation status of each CpG site present within the fragment as reported by the 450K methylation array using ContigExpress software. In all, 35 methylation sites were identified for these miRNAs following bi-directional sequencing (Additional file 1: Table S6).

### Discussion

This study reports the novel association of five miRNAs with DKD, performing validation following the published EWAS and fine-mapping on these miRNAs. Additionally, different sequencing approaches were evaluated to define the genetic and epigenetic architecture of sequences surrounding miRNAs associated with DKD. Comparative analysis between Sanger sequencing and NGS technologies confirmed a 100% concordant call rate for all SNPs identified by both techniques, for duplicate samples, providing reassurance that the original gDNA sequence for miRNAs was unaltered by the cell-line transformation process. Several additional studies have also reported this positive comparison between Sanger and NGS approaches [30–33]. Notably, this is the first report to return results using semiconductor sequencing chemistry and Ion Torrent platforms for top-ranked miRNAs identified from an EWAS. Despite being the gold-standard method, Sanger sequencing is not faultless and has been shown to be inefficient in confirming NGS results for regions with high GC content, and repetitive sequences [31]. NGS methods are reported to be more sensitive and scalable than Sanger sequencing [30, 33, 34].

Regarding DNA methylation, the BST DNA Sanger sequencing analysis mirrored the methylation sites identified through the Infinium Human Methylation 450K analysis. It is advisable to use at least two methods to detect and confirm differential methylation status [35]. Of the five main methods of detecting differential methylation, three were not employed in this study; (1) immunoprecipitation of methylated DNA, (2) methylated DNA capture by affinity purification and (3) reduced representation bisulphite sequencing [36]. The bisulphite-based methods, of which two were employed here, performed optimally in comparison to the others [36].

In conclusion, differential methylation in the five top-ranked miRNAs is associated with DKD and we have provided new details on the genetic architecture surrounding these loci. Targeted NGS compared favourably with Sanger sequencing. Sanger sequencing is costly and time-consuming when assessing many variants, or samples. Targeted NGS provides a robust alternative method, offering more cost-effective and often more sensitive approach.

## Limitations

A potential limitation is that the sequencing data generated with the *Ion PGM™ Template OT2 400 Kit* was not of as high quality as the *Ion PGM™ IC 200 Kit*. Fragments of 400 bp in length had to be prepared and enriched using both the OT2 and ES, not the Ion Chef™ due to chemistry incompatibilities at the time this experiment was undertaken (2014–2015). Both miRNAs with 400 bp fragments, miR-*34A* and miR-*940,* could have primers re-designed to facilitate 200 bp fragments covering the region of interest to provide better coverage of these regions.

## Additional files

**Additional file 1: Table S1.** Characteristics of the individuals present within the HumanMethylation 450K BeadChip array analysis. **Table S2.** Characteristics of the individuals included within the sequencing analysis. **Table S3.** PCR sequences and conditions for miRNA sequencing. **Table S4.** Details of the top-ranked miRNAs identified from 450K Illumina methylation analysis. **Table S5.** SNP results from the fine mapping (gDNA) Sanger sequencing analysis. **Table S6.** Genomic and bisulphite treated DNA sequences for selected miRNAs.

**Additional file 2: Figure S1.** A summary the NGS Ion PGM™ sequencing statistics. a) the sequencing chip loading density, b) the sequencing read lengths presented as a histogram, c) the alignment percentage of sequencing reads to hg19, d) additional sequencing statistics including chip loading, enrichment percentage, comparison of clonal and polyclonal reads, and the percentage of the final library which met the quality threshold for sequencing.

## Abbreviations

Bp: base pairs
BST: bisulphite treated
CKD: chronic kidney disease
CpG: cytosine-phosphate-guanine
DCs: diabetic controls
DKD: diabetic kidney disease
DM: diabetes mellitus
ECACC: European Collection of Authenticated Cell Cultures
eGFR: estimated glomerular filtration rate
ES: Enrichment System
EWAS: epigenome wide association study
gDNA: genomic DNA
GoKinD: Genetics of Kidneys in Diabetes
GWAS: genome wide association study
miRNAs: MicroRNAs
NGS: next generation sequencing
OT2: One Touch™ 2
PCR: polymerase chain reaction
PGM: Personal Genome Machine
QC: quality control
ROI: Republic of Ireland
SNP: single nucleotide polymorphism
T1D: type 1 diabetes
UK: United Kingdom

## References

[1] Byrne C, Caskey F, Dawnay CC, Ford D, Lambie FS, Maxwell H, et al. UK renal registry UK renal registry 19th Annual Report of the renal association. Nephron. 2017;137(suppl1).

[2] Gross JL, De Azevedo MJ, Silveiro SP, Canani H, Caramori ML, Zelmanovitz T (2005). Diabetic nephropathy: diagnosis, prevention, and treatment. Diabetes Care.

[3] United States Renal Data System (2017). 2017 USRDS annual data report: epidemiology of kidney disease in the United States.

[4] Afkarian M, Sachs MC, Kestenbaum B, Hirsch IB, Tuttle KR, Himmelfarb J (2013). Kidney disease and increased mortality risk in type 2 diabetes. J Am Soc Nephrol.

[5] De Ferranti SD, De Boer IH, Fonseca V, Fox CS, Golden SH, Lavie CJ (2014). Type 1 diabetes mellitus and cardiovascular disease: a scientific statement from the American Heart Association and American Diabetes Association. Diabetes Care.

[6] Ahlqvist E, Van Zuydam NR, Groop LC, McCarthy MI (2015). The genetics of diabetic complications. Nat Rev Nephrol.

[7] Wetterstrand K. DNA Sequencing Costs: Data from the NHGRI Genome Sequencing Program (GSP). http://www.genome.gov/sequencingcostsdata. Accessed 1 Jun 2018.

[8] Renkema KY, Stokman MF, Giles RH, Knoers NVAM (2014). Next-generation sequencing for research and diagnostics in kidney disease. Nat Rev Nephrol.

[9] Sandoval J, Heyn HA, Moran S, Serra-Musach J, Pujana MA, Bibikova M (2011). Validation of a DNA methylation microarray for 450,000 CpG sites in the human genome. Epigenetics.

[10] Pidsley R, Zotenko E, Peters TJ, Lawrence MG, Risbridger GP, Molloy P (2016). Critical evaluation of the Illumina MethylationEPIC BeadChip microarray for whole-genome DNA methylation profiling. Genome Biol.

[11] Pezzolesi MG, Poznik GD, Mychaleckyj JC, Paterson AD, Barati MT, Klein JB (2009). Genome-wide association scan for diabetic nephropathy susceptibility genes in type 1 diabetes. Diabetes.

[12] Maeda S, Kobayashi M-A, Araki S-I, Babazono T, Freedman BI, Bostrom MA (2010). A single nucleotide polymorphism within the acetyl-coenzyme A carboxylase beta gene is associated with proteinuria in patients with type 2 diabetes. PLoS Genet.

[13] Sandholm N, Salem RM, McKnight AJ, Brennan EP, Forsblom C, Isakova T (2012). New susceptibility loci associated with kidney disease in type 1 diabetes. PLoS Genet.

[14] Sandholm N, McKnight AJ, Salem RM, Brennan EP, Forsblom C, Harjutsalo V (2013). Chromosome 2q31.1 associates with ESRD in women with type 1 diabetes. J Am Soc Nephrol.

[15] Boger CA, Chen M-H, Tin A, Olden M, Kottgen A, de Boer IH (2011). CUBN Is a gene locus for albuminuria. J Am Soc Nephrol.

[16] Williams WW, Salem RM, McKnight AJ, Sandholm N, Forsblom C, Taylor A (2012). Association testing of previously reported variants in a large case-control meta-analysis of diabetic nephropathy. Diabetes.

[17] McKnight AJ, McKay GJ, Maxwell AP (2014). Genetic and epigenetic risk factors for diabetic kidney disease. Adv Chronic Kidney Dis.

[18] Smyth LJ, Duffy S, Maxwell AP, McKnight AJ (2014). Genetic and epigenetic factors influencing chronic kidney disease. Am J Physiol Renal Physiol.

[19] Maghbooli Z, Larijani B, Emamgholipour S, Amini M, Keshtkar A, Pasalar P (2014). Aberrant DNA methylation patterns in diabetic nephropathy. J Diabetes Metab Disord.

[20] Rna N, Esteller M (2011). Non-coding RNAs in human disease. Nat Rev Genet.

[21] Kato M, Natarajan R (2015). MicroRNAs in diabetic nephropathy: functions, biomarkers, and therapeutic targets. Ann NY Acad Sci.

[22] Cannell IG, Kong YW, Bushell M (2008). How do microRNAs regulate gene expression?. Biochem Soc Trans.

[23] Tüfekci KU, Oner MG, Meuwissen RLJ, Genç S (2014). The role of microRNAs in human diseases. Methods Mol Biol.

[24] He F, Peng F, Xia X, Zhao C, Luo Q, Guan W (2014). MiR-135a promotes renal fibrosis in diabetic nephropathy by regulating TRPC1. Diabetologia.

[25] Kato M, Arce L, Wang M, Putta S, Lanting L, Natarajan R (2011). A microRNA circuit mediates transforming growth factor-Β1 autoregulation in renal glomerular mesangial cells. Kidney Int.

[26] Wang Q, Wang Y, Minto AW, Wang J, Shi Q, Li X (2008). MicroRNA-377 is up-regulated and can lead to increased fibronectin production in diabetic nephropathy. FASEB J.

[27] Public Health England. Culture Collections: About ECACC. https://www.phe-culturecollections.org.uk/collections/ecacc.aspx. Accessed 2 Aug 2018.

[28] Smyth LJ, McKay GJ, Maxwell AP, McKnight AJ (2014). DNA hypermethylation and DNA hypomethylation is present at different loci in chronic kidney disease. Epigenetics.

[29] Benjamini Y, Hochberg Y (1995). Controlling the false discovery rate: a practical and powerful approach to multiple testing. J R Stat Soc Ser B.

[30] Arsenic R, Treue D, Lehmann A, Hummel M, Dietel M, Denkert C (2015). Comparison of targeted next-generation sequencing and Sanger sequencing for the detection of PIK3CA mutations in breast cancer. BMC Clin Pathol.

[31] Baudhuin LM, Lagerstedt SA, Klee EW, Fadra N, Oglesbee D, Ferber MJ (2015). Confirming variants in next-generation sequencing panel testing by sanger sequencing. J Mol Diagn.

[32] Strom SP, Lee H, Das K, Vilain E, Nelson SF, Grody WW (2014). Assessing the necessity of confirmatory testing for exome-sequencing results in a clinical molecular diagnostic laboratory. Genet Med.

[33] Williams EL, Bagg EAL, Mueller M, Vandrovcova J, Aitman TJ, Rumsby G (2015). Performance evaluation of Sanger sequencing for the diagnosis of primary hyperoxaluria and comparison with targeted next generation sequencing. Mol Genet Genomic Med.

[34] D’Argenio V, Esposito MV, Telese A, Precone V, Starnone F, Nunziato M (2015). The molecular analysis of BRCA1 and BRCA2: next-generation sequencing supersedes conventional approaches. Clin Chim Acta.

[35] Chen DP, Lin YC, Fann CSJ (2016). Methods for identifying differentially methylated regions for sequence- and array-based data. Brief Funct Genomics.

[36] Bock C, Tomazou EM, Brinkman A, Müller F (2010). Genome-wide mapping of DNA methylation: a quantitative technology comparison. Nat Biotechnol.

