# Supplementary material for: Validation of differentially methylated microRNAs identified from an epigenome-wide association study; Sanger and next generation sequencing approaches
Source: BMC Res Notes. 2018 Oct 29;11:767. doi: 10.1186/s13104-018-3872-x (PMC6206874; doi:10.1186/s13104-018-3872-x)
Supplement: Supplementary file 1 — Additional file 1: Table S1. Characteristics of the individuals present within the HumanMethylation 450K BeadChip array analysis. Table S2. Characteristics of the individuals included within the sequencing analysis. Table S3. PCR sequences and conditions for miRNA sequencing. Table S4. Details of the top-ranked miRNAs identified from 450K Illumina methylation analysis. Table S5. SNP results from the fine mapping (gDNA) Sanger sequencing analysis. Table S6. Genomic and bisulphite treated DNA sequences for selected miRNAs. [file 13104_2018_3872_MOESM1_ESM.docx]

**Table S1:** Characteristics of the individuals present within the HumanMethylation 450K BeadChip array analysis

| **Characteristics** | **Case** | **Control** |
| --- | --- | --- |
| Number of individuals | 150 | 100 |
| Average Age of T1D diagnosis | 16 years | 12 years |
| Males : Females | 83:67 | 40:60 |
| eGFR > 60mL/min/m^2^ | 0 | 100 |
| eGFR 59-15 mL/min/m^2^ [CKD stages 3-4] | 80 | 0 |
| eGFR <15 mL/min/m^2^ [ESRD] | 70 | 0 |

*Table S1 Abbreviations*: CKD, chronic kidney disease; eGFR, estimated glomerular filtration rate; ESRD, end-stage renal disease; T1D, type 1 diabetes.

**Table S2:** Characteristics of the individuals included within the sequencing analysis

| **Characteristics** | **Case** | **Control** |
| --- | --- | --- |
| Number of individuals | 23 | 23 |
| Average Age of T1D diagnosis | 16 years | 15 years |
| Males : Females | 8:15 | 21:2 |
| eGFR > 60mL/min/m^2^ | 0 | 23 |
| eGFR 59-15 mL/min/m^2^ [CKD stages 3-4] | 8 | 0 |
| eGFR <15 mL/min/m^2^ [ESRD] | 15 | 0 |

*Table S2 Abbreviations*: CKD, chronic kidney disease; eGFR, estimated glomerular filtration rate; ESRD, end-stage renal disease; T1D, type 1 diabetes.

**Table S3:** PCR sequences and conditions for miRNA sequencing

| **Primer Name** | **Primer Sequence** | **PCR Conditions** | **Optimum Annealing Temperature (°C)** | **Fragment Size (bp)** |
| --- | --- | --- | --- | --- |
| *miR-141* Forward Primer | AGGGCTCACCAGGAAGTGT | 95°C – 15 minutes  94°C – 45 seconds  *°C – 45 seconds x35  72°C – 1 minute  72°C – 10 minutes  15°C – 5 minutes  4°C - hold  *optimum annealing temperature as denoted in the following column | 63.4 | 862 |
| *miR-141* Reverse Primer | CTTCCTCATGTTCCCCTTCA |  |  |  |
| *miR-141* Bisulphite Forward | CCTAATAAATCCAAAACCCACAAT |  | 60.7 | 257 |
| *miR-141* Bisulphite Reverse | GGTTTTTAGGGTTTTTTGAAGGTTA |  |  |  |
| *miR-329-2* Forward Primer | CTCAGCTTCAGGGAAGGGCGTTAC |  | 46.7 | 478 |
| *miR-329-2* Reverse Primer | CCAGGCTGCCATCACATTGA |  |  |  |
| *miR-329-2* Bisulphite Forward | AAATTTTATTTTGGAGGTTTTTTGG |  | 56.7 | 374 |
| *miR-329-2* Bisulphite Reverse | ACAACACCCCTTCCCTAAAACTA |  |  |  |
| *miR-34a* Forward Primer | GACCACCACCTTCAGCAACT |  | 48.2 | 468 |
| *miR-34a* Reverse Primer | GAAAGGCCTCAAGAGCAGAA |  |  |  |
| *miR-34a* Bisulphite Forward | AATTGATAAGGAGAAAAAGAGTTAGTATT |  | 56.7 | 255 |
| *miR-34a* Bisulphite Reverse | AATTTCAAACTTACCTAACCAACCC |  |  |  |
| *miR-429* Forward Primer | AAGGACAAGGTGGGTTCAGA |  | 63.4 | 865 |
| *miR-429* Reverse Primer | GATAGCACGGGGTCCACA |  |  |  |
| *miR-429* Bisulphite Forward | TTTATATAGTTAGGTTGGGTTGGGA |  | 53.4 | 252 |
| *miR-429* Bisulphite Reverse | CCAAACAATATTAAACAAAAAACCAA |  |  |  |
| *miR-940* Forward Primer | ATCAGAGGAGGAGCTGCTGA |  | 61.8 | 874 |
| *miR-940* Reverse Primer | GCAGTCACTTAGGCTGCTCA |  |  |  |
| *miR-940* Bisulphite Forward | GTTTTGGTGATTTTGTGGTATTGTT |  | 53.4 | 303 |
| *miR-940* Bisulphite Reverse | AACAAAACCTCCTCCAAAAAATAAA |  |  |  |

*Table S3 Abbreviations***:** bp, base pairs; miR, microRNA.

**Table S4:** Details of the top-ranked miRNAs identified from 450K Illumina methylation analysis

| **miRNA** | **Methylated CpG Site(s)** | **Δβ** | **P_adjusted_ Value** |
| --- | --- | --- | --- |
| *miR-141* | cg18185189 | 0.008 | 2.0x10^-7^ |
| *miR-329-2* | cg06961429 | 0.013 | 1.7x10^-6^ |
| *miR-34A* | cg04329551 | 0.024 | 2.4x10^-7^ |
| *miR-429* | cg12760887 | 0.012 | 3.2x10^-7^ |
| *miR-940* | cg07009125 | 0.019 | 3.3x10^-8^ |

*Table S4 Abbreviations*: CpG, cytosine-phosphate-guanine; miR, microRNA; miRNA, microRNA; Δβ, delta beta (methylation).

**Table S5:** SNP results from the fine mapping (gDNA) Sanger sequencing analysis

| **SNP** | **miRNA** | **Cases** | | **Controls** | | **P value (cases *vs* controls)** | **Reported overall MAF from dbSNP (HapMap-CEU, low coverage panel) (%)** |
| --- | --- | --- | --- | --- | --- | --- | --- |
|  |  | **Genotype Count** | **MAF (%)** | **Genotype Count** | **MAF (%)** |  |  |
| rs141067872 | *miR-329-2* | GG=19  GT=0  TT=0  *4 sequencing fails | T=0 | GG=22  GT=1  TT=0 | T=2.2 | 0.361 | Not provided |
| rs10132943 | *miR-329-2* | GG=17  GA=2  AA=0  *4 sequencing fails | A=5.3 | GG=18  GA=5  AA=0 | A=10.9 | 0.355 | A=5.0 |
| rs7521584 | *miR-429* | TT=0  TG=0  GG=22  *1 sequencing fail | T=0 | TT=0  TG=2  GG=21 | T=4.3 | 0.162 | T=4.1 |
| rs112695918 | *miR-429* | TT=14  TC=3  CC=0  *6 sequencing fails | C=8.8 | TT=23  TC=0  CC=0 | C=0 | 0.040 | C=4.1 |

*Table S5 Abbreviations***:** MAF, minor allele frequency; miR, microRNA; miRNA, microRNA; SNP, single nucleotide polymorphism.

**Table S6:** Genomic and bisulphite treated DNA sequences for selected miRNAs

| ***miR-141* gDNA** **sequence**  cgccccctctcaagagacctcacctggcctgtggccagggtcccctgtagcaactggtgagcg[cg]caccgtagttctctgt**cggccggccctgggtccatcttccagtacagtgttggatggtctaattgtgaagctcctaacactgtctggtaaagatggctcccgggtgggttc**tctcg |
| --- |
| ***miR-141* BST** **DNA** **sequence**  cgTTTTtTtTTaagagaTTtTaTTtggTTtgtggTTagggtTTTTtgtagTaaTtggtgagcg[cg]TaTcgtagttTtTtgt**cggTcggTTTtgggtTTatTttTTagtaTagtgttggatggtTtaattgtgaagTtTTtaaTaTtgtTtggtaaagatggttttcgggtgggttt**tttcg |
| ***miR-329-2* gDNA** **sequence**  cgaagcctgtgctgtattattttgactgatgtcatctgttctactaaccccagtgtccaatc[cg]tatttaacaggacttccaggactgaatgtcaagtttggggaaggaatcagtggtgttcttgtcagtgttacttg**gtggtacctgaagagaggttttctgggtttctgtttctttattgaggacg** |
| ***miR-329-2* BST DNA sequence**  CGAAGTTTGTGTTGTATTATTTTGATTGATGTTATTTGTTTTATTAATTTTAGTGTTTAATT[CG]TATTTAATAGGATTTTTAGGATTGAATGTTAAGTTTGGGGAAGGAATTAGTGGTGTTTTTGTTAGTGTTATTTG**GTGGTATTTGCAGAGAGGTTTTTTGGGTTTTTGTTTTTTTATTGAGGACG** |
| ***miR-34a* gDNA sequence**  tgcgtgaaagggaagcaatgaagtagcgccaactcctcccccgtcccgggcac[cg]tttagggagaggatcagagttctgccagagaatcagaaccagaaggagatatagattaagtttatttcaaggaatcggcttacgtgattgtgggggctggctaggcaagtctgaaatc |
| ***miR-34a* BST DNA sequence**  TGCGTGAAAGGGAAGTAATGAAGTAGCGTTAATTTTTTTTTCGTTTCGGGTAT[CG]TTTAGGGAGAGGATTAGAGTTTTGTTAGAGAATTAGAATTAGAAGGAGATATAGATTAAGTTTATTTTAAGGAATCGGTTTACGTGATTGTGGGGGTTGGTTAGGTAAGTTTGAAATA |
| ***miR-429* gDNA sequence**  cgttctgcagacccaggagggatcagggcagctgcaggaggtggcaggagc[cg]cccccggggccttcccttcacaggccccgcagacaccagcccaggacccggaggccacccacaccac**cgccggccgatgggcgtcttaccagacatggttagacctggccctctgtctaatactgtct** |
| ***miR-429* BST DNA sequence**  CGTTTTGTAGATTTAGGAGGGATTAGGGTAGTTGTAGGAGGTGGTAGGAGT[CG]TTTTCGGGGTTTTTTTTTTATAGGTTTCGTAGATATTAGTTTAGGATTCGGAGGTTATTTATATTAT**CGTCGGTCGATGGGCGTTTTATTAGATATGGTTAGATTTGGTTTTTTGTTTAATATTGTTT** |
| ***miR-940* gDNA sequence**  gccgaagcatccaaca**ccagtgtctgtgaggtgtgggcccggccccaggagcggggcctgggcagccc[cg]tgtgttgaggaaggaaggcagggcccccgctccccgggcctgaccccac**tgcttcagccccctcctgcctgcctacagcctggcctcgagggcccatcaggtgaggggaccccacctgcaccaccccgtcctacctcctggag |
| ***miR-940* BST DNA sequence**  GTCGAAGTATTTAATA**TTAGTGTTTGTGAGGTGTGGGTTCGGTTTTAGGAGCGGGGTTTGGGTAGTTT[CG]TGTGTTGAGGAAGGAAGGTAGGGTTTTCGTTTTTCGGGTTTGATTTTAT**TGTTTTAGTTTTTTTTTGTTTGTTTATAGTTTGGTTTCGAGGGTTTATTAGGTGAGGGGATTTTATTTGTATTATTTCGTTTTATTTTTTGGAG |

**Legend:** CpG sites are highlighted and the target CpG site for each gene noted in brackets. Where shown, the miRNA sequence is in bold.

*Table S6 Abbreviations***:** BST, bisulphite treated; gDNA, genomic DNA; miR, microRNA, miRNAs; microRNAs.
