# Supplementary figures and images for: Validation of differentially methylated microRNAs identified from an epigenome-wide association study; Sanger and next generation sequencing approaches
Source: BMC Res Notes. 2018 Oct 29;11:767. doi: 10.1186/s13104-018-3872-x (PMC6206874; doi:10.1186/s13104-018-3872-x)

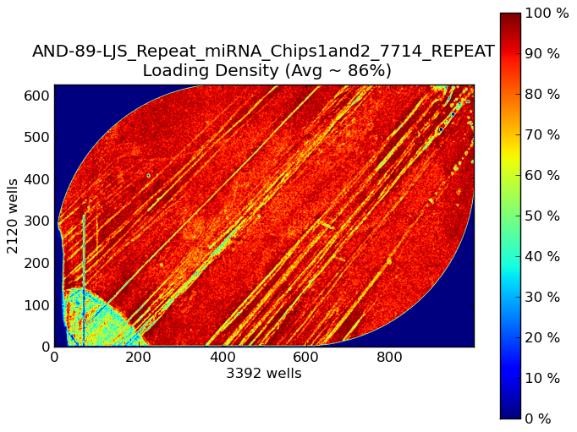

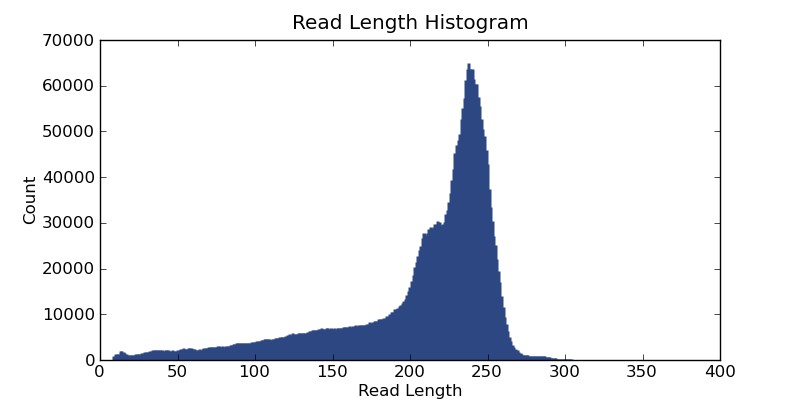

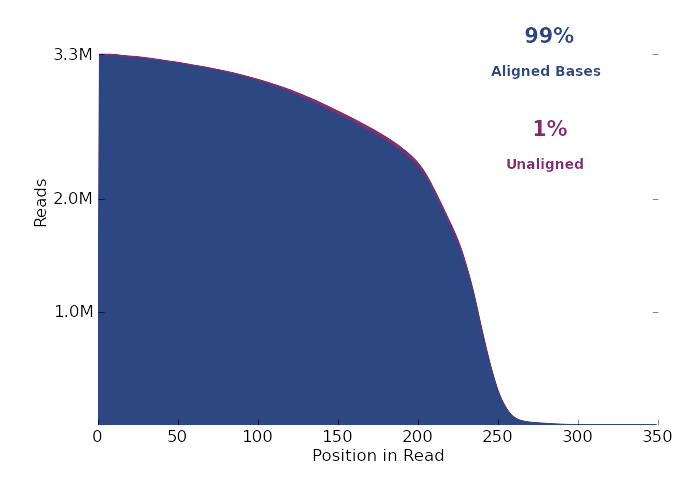

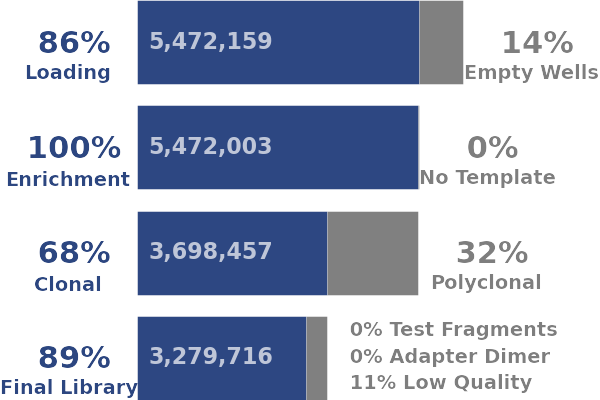

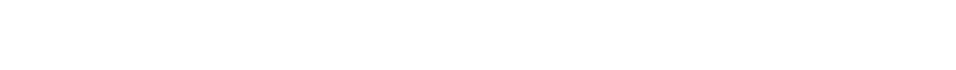


c)

d)


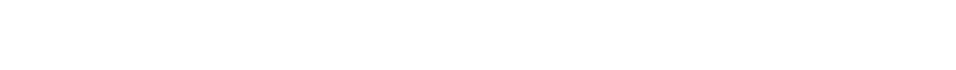


a)

b)

Additional File 2: Figure S1

Supplement: Supplementary file 2 — Additional file 2: Figure S1. A summary the NGS Ion PGM™ sequencing statistics. a) the sequencing chip loading density, b) the sequencing read lengths presented as a histogram, c) the alignment percentage of sequencing reads to hg19, d) additional sequencing statistics including chip loading, enrichment percentage, comparison of clonal and polyclonal reads, and the percentage of the final library which met the quality threshold for sequencing. [file 13104_2018_3872_MOESM2_ESM.docx]
